# Infectious Diseases and Their Outbreaks in Asia-Pacific: Biodiversity and Its Regulation Loss Matter

**DOI:** 10.1371/journal.pone.0090032

**Published:** 2014-02-25

**Authors:** Serge Morand, Sathaporn Jittapalapong, Yupin Suputtamongkol, Mohd Tajuddin Abdullah, Tan Boon Huan

**Affiliations:** 1 Unité de Recherche Animal et Gestion Intégrée des Risques (AGIRs), La Recherche Agronomique pour le Développement/Agricultural Research for Development (CIRAD), Montpellier, France; 2 Institut des Sciences de l’Evolution (ISEM), Centre National de la Recherche Scientifique (CNRS), Institut de Recherche pour le Développement (IRD), Université Montpellier 2, Montpellier, France; 3 Department of Helminthology, Faculty of Tropical Medicine, Mahidol University, Bangkok, Thailand; 4 Department of Veterinary Parasitology, Faculty of Veterinary Sciences, Kasetsart University, Bangkok, Thailand; 5 Center of Excellence on Agricultural Biotechnology, AG-BIO/PERDO-CHE (PERDO/2555-01), Pathum Thani, Thailand; 6 Siriraj Hospital, Faculty of Medical Sciences, Mahidol University, Bangkok, Thailand; 7 Department of Zoology, Universiti Malaysia Sarawak, Kota Samarahan, Sarawak, Malaysia; 8 Department of Civil and Environmental Engineering, Faculty of Engineering, National University of Singapore, Singapore, Singapore; Arizona State University, United States of America

## Abstract

Despite increasing control measures, numerous parasitic and infectious diseases are emerging, re-emerging or causing recurrent outbreaks particularly in Asia and the Pacific region, a hot spot of both infectious disease emergence and biodiversity at risk. We investigate how biodiversity affects the distribution of infectious diseases and their outbreaks in this region, taking into account socio-economics (population size, GDP, public health expenditure), geography (latitude and nation size), climate (precipitation, temperature) and biodiversity (bird and mammal species richness, forest cover, mammal and bird species at threat). We show, among countries, that the overall richness of infectious diseases is positively correlated with the richness of birds and mammals, but the number of zoonotic disease outbreaks is positively correlated with the number of threatened mammal and bird species and the number of vector-borne disease outbreaks is negatively correlated with forest cover. These results suggest that, among countries, biodiversity is a source of pathogens, but also that the loss of biodiversity or its regulation, as measured by forest cover or threatened species, seems to be associated with an increase in zoonotic and vector-borne disease outbreaks.

## Introduction

The Asia-Pacific region, and particularly Southeast Asia, is recognized as a hotspot for biodiversity [1], but is suffering from rapid and extensive erosion of that diversity [2]–[3]. As exemplified by Schipper et al. [4], this region is a major hotspot of mammal diversity at threat, which urges rapid identification of both the drivers of species loss and its consequences for human well-being and health [5]–[6]. Asia-Pacific is also a hotspot for emerging infectious diseases [7]–[8] as illustrated by emergence of Nipah virus, new cholera and dengue variants among others [8]. As biodiversity loss is thought to be a major explanatory factor of the increase in emergence of infectious diseases [7], [9], Asia-Pacific appears a key region where to investigate the links between biodiversity, and its loss, on the patterns of infectious diseases.

Infectious disease (ID) incidence has clearly increased during recent decades [7], [10]. This same time frame has also seen an increase in emergence of infectious diseases (EID) [7]. While some of this increase is undoubtedly due to improvements in disease reporting and surveillance, studies have found that the trends persist even after correction for changes in sampling effort over time [7], [10]. It is emphasized that biodiversity changes through fragmentation and degradation of natural habitats (particularly forested areas) increase the proximity of wildlife to humans, and their domestic animals, and result in increased health risks through increased transmission of zoonotic diseases [11]–[14]. Increasing encroachment of farms on wildlife habitats has increased the overlap between livestock and wild animals, with the consequence that the vast majority of emerging diseases of livestock have been acquired from wild animals [15]. Indeed, escalating human activities as a result of economic development lead to several regional environmental changes, which include altered land use and distributions of domestic animals and wildlife as well as increased international trade. Increased urbanization and agricultural intensification change land use as well as the changes in behaviour and population size or density of humans, and the hosts and vectors, may affect the spread of infectious diseases.

The increased interactions between humans and wildlife resulting from habitat fragmentation are also affected by changes in wildlife species richness and community composition. At the local level, a reduction in biodiversity may lead to an increase in the prevalence and transmission rates of certain vector-borne diseases as reviewed by Keesing *et al.* [9]. Several studies have shown that a reduction in biodiversity at the local level may lead to an increase in the prevalence and transmission rates of certain vector-borne diseases [16] through a “dilution effect” mechanism, suggesting that decreased biodiversity, linked to the loss an ecosystem’s capacity to buffer the spread of pathogens, is a source of both increased prevalence of existing diseases and emergence of new infectious diseases [5], [14]. However, these studies suggest that it is not only the number of species in a given community that may explain the spread of an infectious diseases, but also the composition and structure of the community.

The original level of biological diversity is also important to explain the overall richness of infectious diseases [17]. Bird and mammal species richness (often used as proxies for overall biodiversity) are highest in the tropical zones near the equator, a trend which has also been found for general pathogens and parasites [18]–[19], as well as for human pathogens [17], [20]. The majority of EID are zoonotic diseases, many of wildlife origin, suggesting the importance of biodiversity as a source of EID [7].

Here, we explored the effects of biodiversity on patterns of infectious disease richness and outbreaks in Asia-Pacific. We considered two predictions related to the diversity of infectious diseases and the number of infectious disease outbreaks. First, the richness of ID should increase with increasing biodiversity (after controlling for nation area, population size, climate and surveillance effort), supporting regionally the pattern observed globally by Dunn et al. [17]. Second, the number of ID outbreaks should be related to indices of biodiversity loss, such as forest cover and vertebrate species at threat (after controlling for potential confounding factors), as suggested by several studies (reviewed in [9]).

## Materials and Methods

### Sources of Data

Infectious disease data were obtained from GIDEON (Global Infectious Diseases and Epidemiology Network, www.gideononline.com), which contains information on the presence of human infectious diseases and occurrence of epidemics in each country. This source has regularly been used in comparative studies of pathogen diversity [17], [20] or infectious disease outbreaks [10]. We collected data on socioeconomic, demographic and environmental variables to use as explanatory factors for the disease data. Variables obtained from the World Bank (http://worldbank.org/ddp/home.do) included country area and population, *per capita* gross domestic product (GDP), *per capita* health expenditure, forested surface area and forest as a proportion of each country’s total area. Data on bird and mammal species richness are from Bird Life International (http://birdlife.org/datazone/home) and the International Union for Conservation of Nature (www.iucnredlist.org/initiatives/mammals). *Per capita* healthcare expenditure and the number of diseases for which a survey has been conducted in a country (GIDEON) were used as measures of investigation effort. The increase in health expenditure through time is likely associated with an increase in surveillance detection (potentially also seen in increased surveys) and outbreak reporting. This information was obtained from the Total Economy Database (www.conference-board.org/data/economydatabase/). We restricted our analyses to 28 countries for which sufficient data were available (see Tables S1.1 and S1.2 in File S1 for list of countries and country-level data). The total number of outbreaks was computed from 1950 to 2008 (see also [10]).

### Statistical Analyses

**Figure 1:**
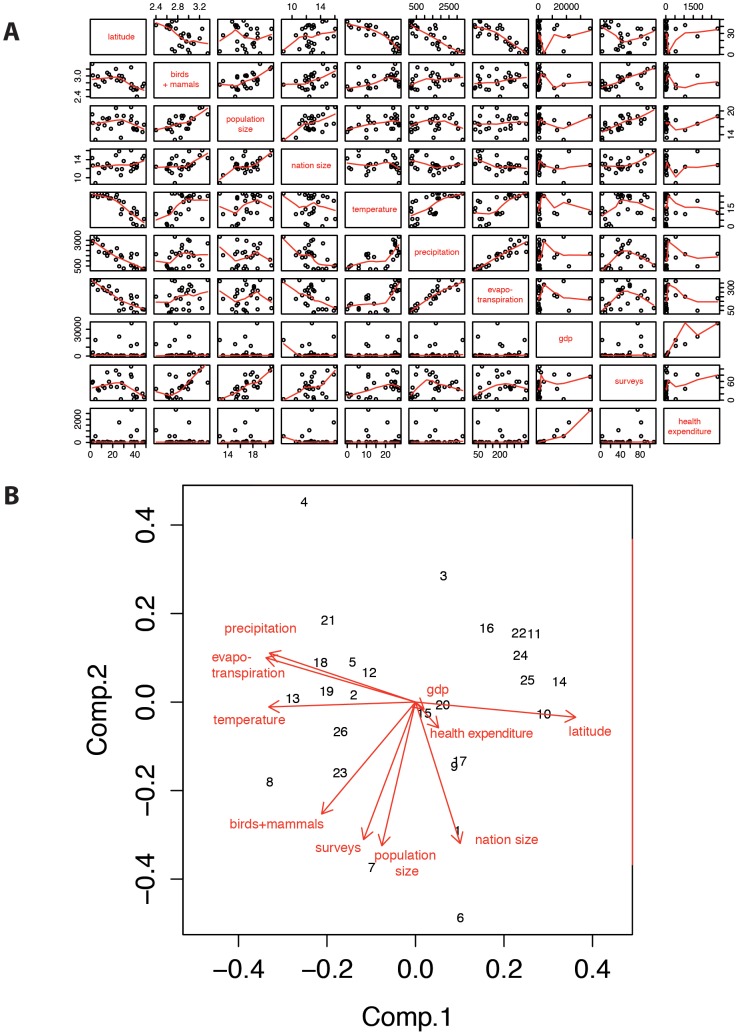
A. Correlation among geographic (latitude, evapotranspiration, nation area size), climate (mean temperature, mean precipitation), and socio-economic variables (population size, GPD *per capita*, health expenditure), and health surveys, potentially linked with the richness infectious diseases and their outbreaks (see raw data in File S1): B. Principal Component Analysis on geographic (latitude, evapotranspiration, nation area size), biodiversity (bird and mammal richness, forest cover, vertebrate species at threat), climate (mean temperature, mean precipitation), and socio-economic variables (population size, GPD *per capita*, health expenditure), and health surveys, potentially linked with the richness infectious diseases. Distributions of variables were normalized using log-transformation or asin-square root transformation (see raw data in File S1).

Distributions of variables were normalized using log-transformation or asin-square root transformation. We performed a principal component analysis (PCA) using the package ade4 (version 1.5–2) implemented in the R freeware programming environment (R Development Core Team, 2012). Correlation analyses and PCA allowed the identification of highly correlated variables such as latitude and temperature, or evapo-transpiration and mean annual precipitation (Fig. 1A & B). This first result permitted to test select the independent variables by removing co-linear variables.

We used general linear models (GLMs) using package lme4 implemented R 2.10 to explain disease richness and number of outbreaks as a function of our explanatory variables. In order to explain to explain the richness in infectious diseases, an initial model included the following explanatory variables (i.e. with the exclusion of latitude and evapo-transpiration, which are strongly correlated respectively with temperature and with precipitation): mean annual temperature, mean annual precipitation, bird and mammal species richness (in log), population size (in log), nation size (in log), number of surveys, GDP and health expenditure. These explanatory variables included the ones selected in the study of Dunn et al. [17], and may allow us to compare our results with the results of these authors. Selection of the best model was done using a step forward procedure based on AICc criterion. Initial and intermediate models are given in File S2 (Table S2.1).

A second initial model, in order to explain the total number of outbreaks, included the following explanatory variables: mean annual temperature, mean annual precipitation, bird and mammal species richness (in log), population size (in log), nation size (in log), number of survey, GDP, health expenditure and richness of infectious diseases. We added also two other variables that represent the importance of impacts on biodiversity, the number of threatened bird and mammal species and the percent forest cover of the country. Additionally, we separated the disease outbreaks into three types: total, zoonotic, and vector-borne, and modelled each category. Selection of the best models was done using a step forward procedure based on AICc criterion. Initial and intermediate models are given in File S2 (Tables S2.2, 3 & 4). We controlled for the effect of multicollinearity among explanatory variables using a test of variance inflation factors (VIFs) (values VIFs less than 10, “rules of thumb”, indicate that explanatory variables were uncorrelated with other for all the models tested).

In order to illustrate graphically the effects of patterns of biodiversity and biodiversity loss on the richness and total number of outbreaks, we used the residuals of the selected regression models.

## Results

### Infectious Diseases Richness

**Figure 2:**
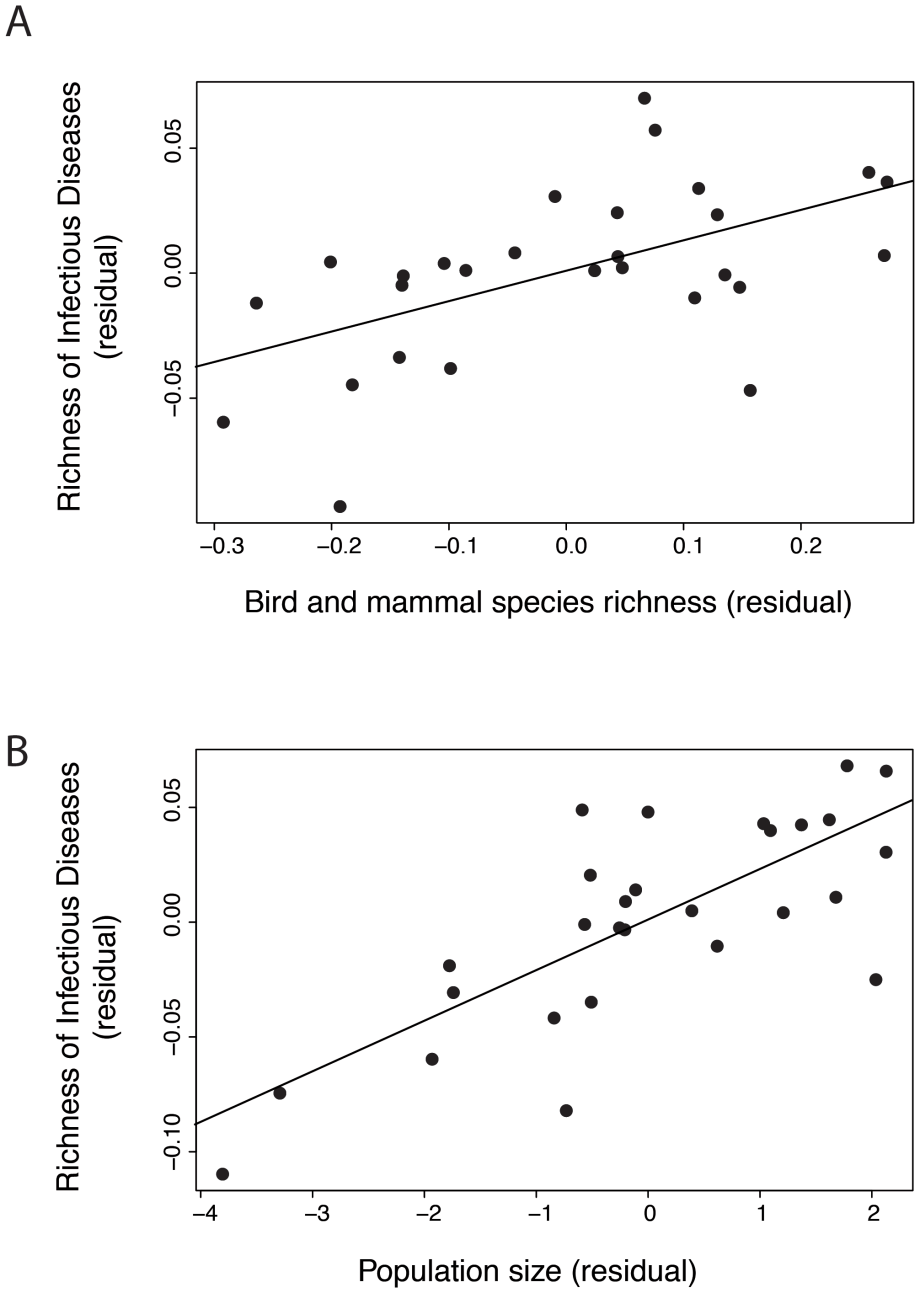
Partial relationships (partial correlation using residuals of the best models selected in Table 1) between the richness of infectious diseases and (A) the richness of bird and mammal species and (B) population size (partial correlation of the best GLM selected in Table 1).

The best model showed that the richness of infectious diseases is positivey linked to temperature (i.e. climate), population size and richness in bird and mammal species (see File S1, Table S2.1, Table 1). A country with high biodiversity, in terms of birds and mammals, and a large population, hosts a greater diversity of infectious diseases as represented graphically (Fig. 2A &B).

**Table 1:** Summarized results for a General Linear Model explaining the richness of infectious diseases in Asia-Pacific.

Dependent variable	Explanatory variables	Effect	F (P)	VIF	R^2^ (P)
Richness of infectious diseases	Temperature	+0.001 (0.97)	14.3 (<0.001)	1.37	
	Population size	+0.02 (<0.0001)	32.4 (<0.0001)	1.99	
	Richness of birds and mammals	+0.12 (0.0036)	66.4 (<0.0001)	1.54	
					0.82 (<0.0001)

Initial variables were: nation area, population size, richness in bird and mammal species, mean temperature, mean precipitation, surveys, GDP and health expenditure. Selection of the model was based on AIC criterion. The multicollinearity among independent variables is assessed by the variance inflated factor (VIF).

### ID Outbreaks and Biodiversity

**Figure 3:**
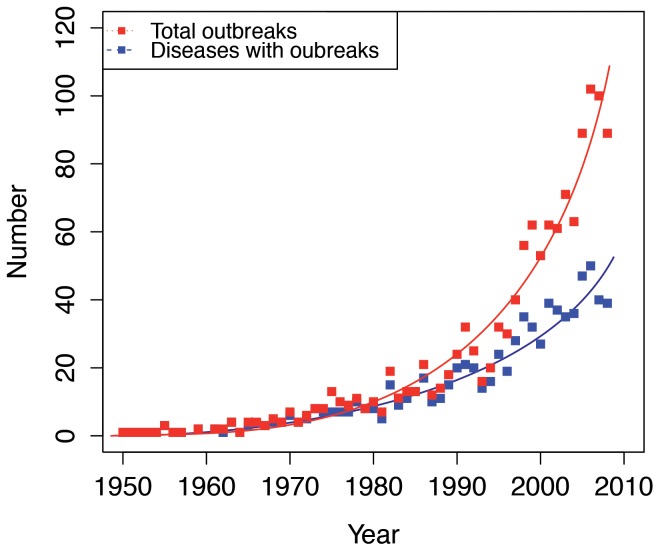
Increase in total outbreaks and total number of infectious diseases causing outbreaks since 1950 in Asia-Pacific countries.

Using the GIDEON data base, and over the period from 1950 to 2008, 124 different diseases with epidemics were identified in Asia-Pacific countries. An increase in both the number of outbreaks and the number of different diseases causing outbreaks through time was observed (Fig. 3).

Three models were created to explain the number of total infectious diseases, the total number of zoonotic infectious diseases and the total number of vector-borne diseases with outbreaks (for complete model selection process see File S2, Tables S2.2, S2.3, S2.4).

**Figure 4:**
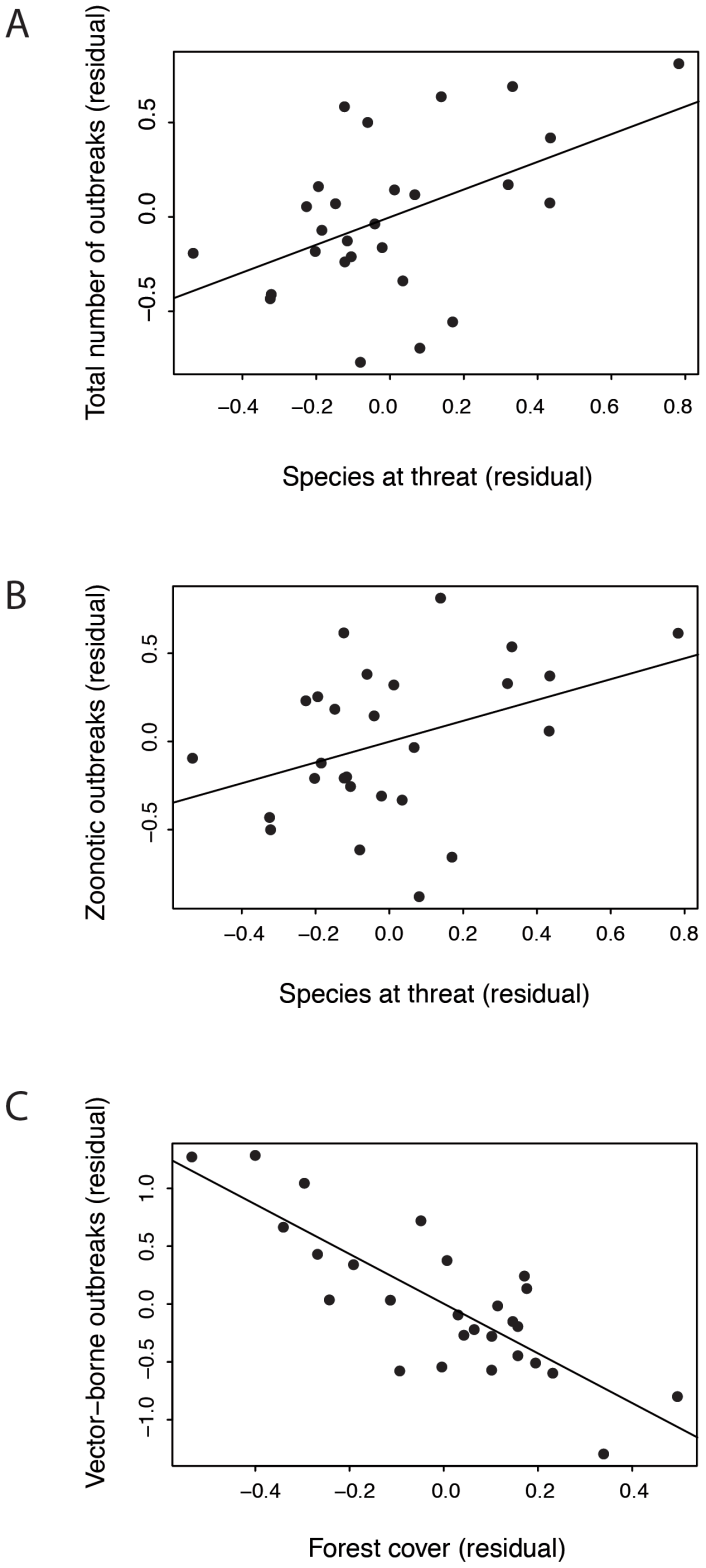
Relationships between the number of diseases causing outbreaks and biodiversity indices in Asia-Pacific countries (partial correlations of the best GLM selected in Table 2). A. Total number of infectious diseases with outbreaks and number of threatened vertebrate species. B. Total zoonotic diseases with outbreaks and number of threatened vertebrate species. C. Total number of vector-borne diseases with outbreaks and forest cover.

The number of total infectious diseases with outbreaks was found to be positively associated with temperature, forest cover, health expenditure, surveys, number of threatened bird and mammal species and population size (Table 2a). The positive association of outbreaks with the number of threatened vertebrate species is represented graphically using the residual variations of the selected model (Fig. 4A).

**Table 2:** Summarized results for a General Linear Model explaining the number of infectious diseases with epidemics (total, zoonotic and vector-borne) in Asia-Pacific.

Dependent variable	Explanatory variables	Effect (P)	VIF	F (P)	R^2^ (P)
a. number of infectious diseaseswith outbreaks	Mean temperature	−0.02 (0.20)	2.27	0.2 (0.69)	
	Forest cover	−0.84 (0.037)	2.08	0.36 (0.55)	
	Health expenditure	+0.00005 (0.003)	1.62	11.3 (0.003)	
	Surveys	+0.02 (0.002)	3.93	11.9 (0.003)	
	Number of threatened species	+0.72 (0.022)	2.24	98.8 (<0.0001)	
	Population size	+0.21 (0.016)	3.69	104.2 (<0.0001)	0.92 (<0.0001)
b. number of zoonotic diseaseswith outbreaks	Mean temperature	−2.47 (0.12)	2.11	0.02 (0.89)	
	Forest cover	−0.90 (0.071)	1.95	0.03 (0.86)	
	Health expenditure	+0.0004 (0.013)	1.63	7.4 (0.01)	
	Surveys	+0.02 (0.009)	3.77	10.3 (0.004)	
	Number of threatened species	+0.59 (0.08)	2.27	64.5 (<0.0001)	
	Population size	+0.24 (0.01)	3.60	86.7 (<0.0001)	0.89 (<0.0001)
c. number of vector-borne diseaseswith outbreaks	Health expenditure	+0.0007 (0.001)	1.40	21.8 (<0.0001)	
	Richness of Infectious Diseases	+1.2 (0.0003)	6.03	6.8 (0.02)	
	Forest cover	−2.1 (<0.0001)	1.71	23.2 (<0.0001)	
	Richness of birds and mammals	+2.1 (0.003)	2.97	81.4 (<0.0001)	
	Population size	−0.11 (0.21)	5.16	53.9 (<0.0001)	0.90 (<0.0001)

Initial variables were: nation area size, population size, richness of bird and mammal species, number of threatened vertebrate species, proportion of forest, mean temperature, mean precipitation, surveys, GDP and health expenditure. Selection of the best models was based on AIC criterion (see all models in File S2). Selected variables are ranked by increasing contribution to the model (F value). The multicollinearity among independent variables is assessed by the variance inflated factor (VIF).

The total number of zoonotic diseases with epidemics was found associated with mean temperature, forest cover, health expenditure, surveys, number of threatened bird and mammal species and population size (Table 2b). The positive correlation of zoonotic outbreaks with threatened species is represented graphically using the residual variations of the selected model (Fig. 4B).

The total number of vector-borne diseases with epidemics was correlated positively with health expenditure, richness of infectious diseases, richness of bird and mammal species, and population size and negatively with forest cover and population size (Table 2c). The negative association between forest cover and vector-borne outbreaks is represented graphically using the residual variations of the selected model (Fig. 4C).

## Discussion

Although the number of epidemics has increased worldwide [7], [10], the number and kinds of diseases obviously differ greatly among regions. Hence, recent studies have justified our choice to focus on the Asia-Pacific region relating to biodiversity richness and loss [2]–[4] and emerging and remerging diseases [8].

The results of our analyses seem to support that effectively biodiversity if a source of infectious diseases in this region, but that biodiversity loss may have increased the number of outbreaks of infectious diseases over the last decades.

### Biodiversity and the Richness of Infectious Diseases

Our study is a step forward in the analysis of the relationship between biodiversity and infectious diseases. Several studies have emphasized the need to preserve vertebrate biodiversity and community composition in order to significantly reduce the risk of emergence [9], [16], [21]–[22] and while we agree with that assessment when considering incidence or outbreaks, our results suggest that conversely, pathogen richness may in fact be enhanced by a high level of biodiversity. The results presented here on the determinants of infectious disease richness, although focused on Asia-Pacific countries, agree with analyses carried out in Europe [23] and worldwide [17]. Dunn et al. [17] analyzed the diversity of human parasites by nation and found that pathogen diversity increased with human population size and the diversity of birds and mammals in a nation. We found the same result at the scale of Asia-Pacific. Biodiversity appears to be associated with the diversity of human infectious diseases. A country with high bird and mammal diversity likely also harbours a high number of vectors and reservoirs, which constitute the essential elements for the transmission of infectious diseases.

### The Drivers of Disease Outbreaks: Biodiversity Matters

Our dataset showed a dramatic increase in the number of outbreaks, both total number and number of diseases presenting outbreaks, reported in Asia-Pacific over the last sixty years. This is in accordance with observations made in Europe [10], [23] as well as with the global trend for EID [7]. It is important to note, however, that the number of outbreaks is correlated with increased healthcare expenditures, which may reflect improvements in disease detection (and better recording of diseases). Indeed, the number of surveys, another measure of sampling effort, was also found positively correlated with the number of diseases with outbreaks.

Our statistical models have identified several potential explanatory factors for the increase in outbreaks. The inclusion of mean temperature underscores the importance of climate in the occurrence of epidemics.

For each of the three investigated types of disease, outbreaks are correlated with either biodiversity at threat and/or proportion forest cover, a proxy measurement for biodiversity. However, it should be noted that the use of the number of threatened species the forest cover may describe quite poorly the dynamics of biodiversity loss and more accurate and precise indicators of biodiversity loss should be used. New accurate and precise indicators that integrate changes in ecosystem functions and are comparable among nations and scientifically sound are necessary (see [24]).

Our results seem to support the hypothesis of the potential role of biodiversity as a buffer of pathogen spread. As shown at the local scale, biodiversity could reduce outbreaks of vector-transmitted diseases through a dilution effect [21]–[22]. Biodiversity can be linked to reduced pathogen transmission through a dilution effect [9], [21], [25], which may occur when the addition of one or more host species to a host community is linked to “wasted transmissions” and decreased pathogen persistence [9], [21]. The initial proposed mechanism was a “direct” dilution effect with an effective lost of infective stages in “wrong” arthropod vectors (i.e. Lyme disease, West Nile fever). However, a recent meta-analysis has shown heterogeneous links between host biodiversity and disease [26], suggesting that disease risk is more likely a local phenomenon that relies on the specific composition of reservoir hosts and vectors, and their ecology, rather than patterns of species biodiversity. Indeed, the expected links between host diversity and parasite transmission may be quite complex. Other mechanisms were proposed for directly transmitted diseases, often named “indirect dilution effects”, where higher host diversity may lead to a reduction of the susceptible host population size via interspecific competition, which in turn will decrease the transmission. Hence, the local conditions of host species richness and composition in reservoir hosts may be the determining factors as to whether an increased number of potential hosts will divert transmission from highly competent reservoir host species and results in fewer new infections or not.

Our results obtained at a regional scale seem to agree with many of these previous observations made at the local level, which showed an increase in the incidence of infectious diseases with a reduction in biodiversity [21], [25].

### Implications for Human Well-being

The ecosystem approach has been endorsed by the Millennium Ecosystem Assessment [27] as a conceptual framework. This approach defines ecosystem services as the benefits that people obtain from healthy ecosystems. One such ecosystem service is the regulation of human and animal diseases, a benefit largely due to positive effects of biodiversity on disease regulation [28]. Bond et al. [29] showed the benefit effect of biodiversity on human health, where vector-borne and parasite diseases have affected economic development. Our results follow the conclusion of this study and may suggest that although biodiversity is a source of pathogens, well-preserved biodiversity could act an insurance against outbreaks. However, much work is necessary to confirm these results at regional and local levels where land planning can integrate the management of biodiversity as a way to improve human health [29].

### Implication for Epidemiological Surveillance

The results presented here have one major implication for surveillance. The effect of biodiversity on infectious diseases suggests that higher resolution surveillance, both geographic and temporal, is needed. With better surveillance one could, for example, study the statistical relationships between the changing uses of land (fragmentation of landscape, increasing or reducing forest areas) and the occurrence of epidemics in order to investigate different scenarios for the effects of changes in biodiversity on infectious risks [30].

## Supporting Information

File S1 (DOC)

File S2 (DOC)
