# Supplementary material for: Infectious Diseases and Their Outbreaks in Asia-Pacific: Biodiversity and Its Regulation Loss Matter
Source: PLoS One. 2014 Feb 25;9(2):e90032. doi: 10.1371/journal.pone.0090032 (PMC3934982; doi:10.1371/journal.pone.0090032)
Supplement: File S1 — (DOC) [file pone.0090032.s001.doc]

**Supplementary Information 1. Table S1.1 Raw data for each country of the variables used in the study**

| Country | nation size | population | elev | temp | precip | Tot Dis | TO | VBO | ZO | sur-vey | health exp | GDP | b+m | forest | threat |
| --- | --- | --- | --- | --- | --- | --- | --- | --- | --- | --- | --- | --- | --- | --- | --- |
| Afghanistan | 646,212 | 2,3630,320 | 1885 | 12 | 313 | 201 | 23 | 9 | 18 | 9 | 11 | - | 509 | 2.1 | 5 |
| Australia | 7,662,592 | 19,153,000 | 272 | 21 | 472 | 211 | 265 | 43 | 176 | 82 | 1,729 | 21,768 | 1,017 | 20.2 | 22 |
| Bangladesh | 135,887 | 140,766,909 | 85 | 26 | 2227 | 207 | 31 | 6 | 24 | 42 | 9 | 335 | 744 | 11.3 | 9 |
| Bhutan | 38303,91 | 561,175 | 2220 | 10 | 1550 | 191 | 4 | 0 | 3 | 3 | 53 | 762 | 712 | 78.4 | 7 |
| Brunei | 6,733 | 333,471 | 479 | 27 | 3341 | 191 | 3 | 0 | 1 | 3 | 541 | 17,996 | 558 | 75.3 | 9 |
| Cambodia | 182,318 | 12,759,811 | 126 | 27 | 1780 | 213 | 8 | 1 | 4 | 39 | 17 | 286 | 677 | 65.4 | 13 |
| China | 9,344,594 | 1,262,645,000 | 1839 | 6 | 562 | 246 | 195 | 35 | 127 | 108 | 44 | 949 | 1,772 | 19.0 | 12 |
| India | 3,151,251 | 1,015,923,000 | 622 | 24 | 1114 | 246 | 392 | 122 | 288 | 95 | 21 | 453 | 1,595 | 22.0 | 13 |
| Indonesia | 1,697,375 | 205,280,270 | 367 | 25 | 2701 | 238 | 53 | 33 | 48 | 56 | 16 | 804 | 2,237 | 54.9 | 16 |
| Japan | 358,359 | 126,870,000 | 438 | 11 | 1694 | 215 | 371 | 14 | 208 | 75 | 2,827 | 36,789 | 582 | 68.2 | 15 |
| Kazakhstan | 2,707,851 | 14,883,600 | 387 | 6 | 248 | 209 | 36 | 22 | 34 | 9 | 51 | 1,229 | 597 | 1.2 | 8 |
| Kyrgyzstan | 196,276 | 4,915,300 | 2988 | 1 | 423 | 206 | 24 | 11 | 17 | 11 | 13 | 279 | 405 | 4.5 | 6 |
| Lao PDR | 231,130 | 5,403,262 | 710 | 23 | 1888 | 213 | 15 | 3 | 12 | 43 | 10 | 321 | 906 | 71.6 | 9 |
| Malaysia | 323,151 | 23,273,615 | 420 | 25 | 2945 | 231 | 63 | 16 | 37 | 59 | 128 | 4,030 | 1,038 | 65.7 | 18 |
| Mongolia | 1,558,417 | 2,389,197 | 1528 | 0 | 197 | 200 | 11 | 5 | 8 | 21 | 22 | 456 | 494 | 7.5 | 7 |
| Myanmar | 656,424 | 46,609,819 | 702 | 23 | 1994 | 218 | 19 | 10 | 17 | 25 | 3 | - | 1,311 | 53.4 | 8 |
| Nepal | 147,403 | 24,431,756 | 2565 | 14 | 1377 | 203 | 33 | 8 | 27 | 55 | 12 | 225 | 996 | 27.2 | 6 |
| New Zealand | 249,004 | 3,857,800 | 388 | 10 | 1652 | 189 | 83 | 1 | 48 | 36 | 1,055 | 13,336 | 252 | 31.4 | 25 |
| Pakistan | 871,274 | 138,080,000 | 1018 | 20 | 264 | 217 | 51 | 18 | 36 | 40 | 15 | 536 | 802 | 2.7 | 9 |
| Papua New Guinea | 422,882 | 5,387,613 | 668 | 24 | 3060 | 206 | 14 | 5 | 8 | 35 | 26 | 654 | 976 | 66.5 | 12 |
| PDR Korea | 126,960 | 22,858,710 | 599 | 6 | 1006 | 210 | 3 | 0 | 2 | 3 | - | - | 415 | 57.6 | 9 |
| Philippines | 232,173 | 77,689,369 | 442 | 25 | 2549 | 222 | 43 | 10 | 33 | 45 | 33 | 977 | 758 | 23.9 | 19 |
| Republic of Korea | 91,306 | 47,008,000 | 282 | 11 | 1321 | 217 | 70 | 4 | 47 | 82 | 543 | 11,347 | 445 | 63.7 | 10 |
| Sri Lanka | 61,956 | 18,713,711 | 228 | 27 | 1874 | 212 | 30 | 8 | 22 | 31 | 33 | 873 | 493 | 33.2 | 19 |
| Tajikistan | 146,659 | 6,172,891 | 3186 | 2 | 561 | 203 | 16 | 5 | 11 | 17 | 6 | 139 | 395 | 2.9 | 6 |
| Thailand | 506,001 | 62,346,822 | 287 | 26 | 1496 | 235 | 107 | 24 | 77 | 89 | 67 | 1,968 | 1,238 | 37.2 | 14 |
| Turkmenistan | 476,077 | 4,501,727 | 230 | 15 | 165 | 203 | 5 | 3 | 4 | 3 | 45 | 645 | 465 | 8.8 | 8 |
| Uzbekistan | 445,830 | 24,650,000 | 420 | 12 | 194 | 203 | 14 | 2 | 11 | 9 | 32 | 558 | 436 | 7.6 | 7 |
| Vietnam | 333,168 | 77,635,400 | 398 | 23 | 1794 | 223 | 41 | 12 | 26 | 59 | 22 | 402 | 1,115 | 37.7 | 12 |

**Table S1.2. Description of variables and their sources**

| Variable | Description | Source |
| --- | --- | --- |
| nation size | Surface area (km2) | http://www.cid.harvard.edu/ciddata/geographydata.htm |
| population | Population size in 2000 | World Bank |
| elev | Elevation, in mean meter above sea level | http://www.cid.harvard.edu/ciddata/geographydata.htm |
| temp | Annual mean temperature in degree Celsius | http://www.worldclim.org/ |
| precip | Annual precipitation in mm | http://www.worldclim.org/ |
| Tot Dis | Number of pathogens endemic or potentially endemic to a country (1950-2009) | GIDEON |
| TO | The sum of all outbreaks listed for a country (can include multiple for a disease) (1950-2009) | GIDEON |
| VBO | The sum of all vector-borne diseases that had an outbreak in that country (1950-2009) | GIDEON |
| ZO | The sum of all zoonotic outbreaks listed for a country (can include multiple for a disease) (1950-2009) | GIDEON |
| survey | The number of surveys listed for a country (1950-2009), as a measure of the investigation on infectious diseases’ occurrences in that country | GIDEON |
| health exp | Health expenditure *per capita* in 2000, current USD | World Bank |
| gdp | GDP *per capita* in 2000, current USD | World Bank |
| b+m | Number of bird species found in a country  +Number of mammal species found in a country | [http://www.birdlife.org/](http://www.birdlife.org/datazone/species/search)  http://www.iucnredlist.org/ |
| forest | Forest in 2000, in % of land area | World Bank |
| threat | Number of bird and mammal species at threat of extinction | IUCN Red List |
