# Supplementary material for: Infectious Diseases and Their Outbreaks in Asia-Pacific: Biodiversity and Its Regulation Loss Matter
Source: PLoS One. 2014 Feb 25;9(2):e90032. doi: 10.1371/journal.pone.0090032 (PMC3934982; doi:10.1371/journal.pone.0090032)
Supplement: File S2 — (DOC) [file pone.0090032.s002.doc]

**Supplementary Information 2**

**Table S2.1 Factors explaining the richness of infectious diseases.**

Comparison of models used to test the effect of several independent variables tmean (mean annual temperature), mean annual precipitation (precip), bird and mammal species richness (in log) (bm), population size (in log) (pop)+nation size (in log) (area), number of survey (surveys), gdp, health expenditure (hexp) on the diversity of infectious diseases in Asia-Pacific (see supplementary information 2 for data at the level of each country). Models are ranked from the least to the most supported according to corrected Akaike information criteria (AIC).

| Model ranks | AIC |
| --- | --- |
| precip+bm +pop | -108.7 |
| tmean + bm + pop | -108.7 |
| tmean+precip+bm+pop | -106.7 |
| tmean+precip+bm+pop+surveys | -106.0 |
| precip + evap + bm + pop + area | -106.0 |
| mean+precip+bm+pop+surveys+hexp | -103.6 |
| tmean+precip+bm+pop+area+surveys+hexp | -102.5 |
| mean+precip+bm+pop+hexp | -100.6 |
| tmean+precip+bm+pop+surveys+gdp+hexp | -96.9 |
| tmean+precip+bm+pop+area+surveys+gdp+hexp | -96.7 |

**Table S2.2 Factors explaining the number of outbreaks of infectious diseases.**

Comparison of models used to test the effect of several independent variables tmean (mean annual temperature), mean annual precipitation (precip), bird and mammal species richness (in log) (bm), population size (in log) (pop)+nation size (in log) (area), number of survey (surveys), gdp, health expenditure (hexp), richness in infectious diseases (path), forest cover (asin square root of forest percentage) (forest) and bird and mammal species at threat (threat) on the number of total events of outbreaks of infectious diseases in Asia-Pacific (see supplementary 2 for data at the level of each country). Models are ranked from the least to the most supported according to corrected Akaike information criteria (AIC).

| Model ranks | AIC |
| --- | --- |
| tmean + threat + pop + forest + surveys + hexp | 36.9 |
| threat + pop + forest + area + surveys + hexp | 37.6 |
| tmean + threat + pop + forest + area + surveys + hexp | 38.9 |
| tmean + bm + threat + pop + forest + area + surveys + hexp | 40.2 |
| tmean + bm + threat + pop + path + forest + area + surveys + hexp | 41.7 |
| tmean + precip + bm + threat + pop + path + forest + area + surveys + hexp | 43.0 |
| tmean + precip + bm + threat + pop + path + forest + area + surveys + gdp + hexp | 43.1 |
| tmean + precip + bm + threat + pop + path + forest + area + surveys + gdp + hexp | 45.1 |

**Table S2.3 Factors explaining the number of outbreaks of zoonotic diseases.**

Comparison of models used to test the effect of several independent variables tmean (mean annual temperature), mean annual precipitation (precip), bird and mammal species richness (in log) (bm), population size (in log) (pop)+nation size (in log) (area), number of survey (surveys), gdp, health expenditure (hexp), richness in infectious diseases (path), forest cover (forest)(asin square root of forest percentage) and bird and mammal species at threat (threat) on the number of total number of outbreaks of zoonotic diseases in Asia-Pacific (see supplementary information 2 for data at the level of each country). Models are ranked from the least to the most supported according to corrected Akaike information criteria (AIC).

| Model ranks | AIC |
| --- | --- |
| tmean + threat + pop + forest + surveys + hexp | 41.9 |
| tmean+pop+forest+surveys+hexp | 44.1 |
| tmean+threat+pop+surveys+hexp | 44.4 |
| tmean+precip+threat+pop+forest+area+surveys+hexp | 44.9 |
| tmean+precip+threat+pop+path+forest+area+surveys+hexp | 46.2 |
| tmean + precip + bm + threat + pop + path + forest + area + surveys + gdp+hexp | 47.8 |
| tmean + precip + bm + threat + pop + path + forest + area + surveys + gdp+hexp | 48.1 |
| tmean + precip + bm + threat + pop + path + forest + area + surveys + gdp | 49.2 |

**Table S2. 4 Factors explaining the number of outbreaks of vector-borne diseases.**

Comparison of models used to test the effect of several independent variables tmean (mean annual temperature), mean annual precipitation (precip), bird and mammal species richness (in log) (bm), population size (in log) (pop)+nation size (in log) (area), number of survey (surveys), gdp, health expenditure (hexp), richness in infectious diseases (path), forest cover (forest)(asin square root of forest percentage) and bird and mammal species at threat (threat) on the number of total number of outbreaks of vector-borne diseases in Asia-Pacific (see supplementary information 2 for data at the level of each country). Models are ranked from the least to the most supported according to corrected Akaike information criteria (AIC).

| Model ranks | AIC |
| --- | --- |
| bm+pop+path+forest+hexp | 35.0 |
| tmean+bm+pop+path+forest+hex | 36.9 |
| tmean+bm+threat+pop+path+forest+hexp | 38.5 |
| tmean+bm+threat+pop+path+forest+area+hexp | 38.9 |
| tmean + bm + threat + pop + forest + area + surveys + hexp | 40.4 |
| tmean + bm + threat + pop + path + forest + area + surveys + hexp | 41.7 |
| tmean + precip + bm + threat + pop + path + forest + area + surveys + hexp | 42.7 |
| tmean+bm+threat+pop+path+forest+area+surveys+hexp | 42.33 |
| tmean + precip + bm + threat + pop + path + forest + area + surveys + gdp + hexp | 42.8 |
| tmean + precip + bm + threat + pop + path + forest + area + surveys + gdp | 45.6 |
